# Comparison of the Efficacy of Tolterodine versus Oxybutynin in the Treatment of Children with Desmopressin-Resistant Enuresis: A Randomized Controlled Clinical Trial

**DOI:** 10.4314/ejhs.v33i4.7

**Published:** 2023-07

**Authors:** Neda Ezodin, MahboubehJafari Sarouei, Mohamad Khademlo, Sevda Hashemi Milani, Sahar Yousefi, Hamid Mohammadjafari

**Affiliations:** 1 Department of pediatrics, Mazandaran University of Medical Sciences, Sari Iran; 2 Pediatric infectious diseases research center, communicable Diseases institute, Mazandaran University of Medical Sciences, Sari Iran; 3 MazandaranUniversity of Medical Sciences, Sari Iran; 4 Department of pediatrics, Mazandaran University of Medical Sciences, Sari Iran; 5 Pediatric infectious diseases research center, communicable Diseases institute, Mazandaran University of Medical Sciences, Sari Iran

**Keywords:** Enuresis, Oxybutynin, Tolterodine, Desmopressin

## Abstract

**Background:**

Enuresis, defined as involuntary nocturnal urination without any underlying organic disorder in a child expected to control urination, poses a common problem. This study evaluated the effectiveness of Tolterodine and Oxybutynin in children presenting with primary desmopressin-resistant enuresis.

**Materials and Methods:**

A randomized clinical trial was undertaken involving 68 participants aged between 5 and 16 years, all suffering from primary enuresis. These patients were randomly assigned to one of two treatment groups for a three-month period: Group 1, treated with Oxybutynin and Desmopressin, and Group 2, treated with Tolterodine and Desmopressin. Data on demographics, clinical and laboratory findings, and subjective responses to treatment were gathered. The response was measured based on the frequency of wetting incidents per night and week and compared with pre-treatment data.

**Results:**

Patients were divided into two groups (30 patients in Group 1 and 38 patients in Group 2). The mean age of the patients was 88.97±27.09 months. In the first treatment group, 6 out of 30 patients (20%) experienced a complete treatment response, as did 5 out of 38 patients (13.2%) in the second treatment group. This difference between the groups was not statistically significant. Seven patients (23%) in the Oxybutynin group and 13 patients (34%) in the Tolterodine group reported a lack of response to treatment, a difference that also lacked statistical significance.

**Conclusion:**

For patients resistant to Desmopressin, the addition of anticholinergic drugs elicited a significant response in over half of the patients. However, no benefit was observed in using either Oxybutynin or Tolterodine in the treatment of Desmopressin-resistant enuresis.

## Introduction

Urinary incontinence, defined as involuntary nocturnal urination devoid of any physical ailments in a child expected to exercise urinary control, presents a multifaceted problem with significant clinical implications (1,2). It is rare to see urinary control before the age of 1.5 years. By the age of 4.5 years, 20% of children annually achieve urinary control. The prevalence of nocturnal incontinence is 15% at five years of age and declines to 1% in adolescents aged 15 years and older (3). Enuresis constitutes a complex issue for primary school children, posing a challenge for both the child and the parents (4). If left unaddressed, it can lead to behavioral issues in children, parental anxiety, and even affect the child's social interactions in adulthood (4). Recent research has identified three central mechanisms for enuresis: nocturnal polyuria, overactive detrusor muscle, and an elevated arousal threshold (5). It has been found that many children with enuresis struggle with the natural nocturnal increase in vasopressin secretion, leading to a significant rise in urine output (6 - 8).

Various treatments are available for enuresis. The most effective methods include tricyclic antidepressants, oxybutynin, desmopressin, conditioning therapy, and behavioral therapy (9). Desmopressin boasts a success rate of 60-70%, though it has a recurrence rate of 20-40% (9, 10). Behavioral interventions encompass alarm usage, learning, star charts, reward systems, bladder control exercises, fluid management, and wake-up programs (11). These methods either condition awareness or enhance bladder reservoir function (11). Although these treatments have proven effective, their recurrence rate remains high. Furthermore, it is critical to provide suitable treatment for patients resistant to conventional therapies (12).

The first-line pharmaceutical intervention for children with enuresis resistant to behavioral therapy is nasal or oral desmopressin. The second-line treatment for enuretic children unresponsive to desmopressin consists of anticholinergic drugs (3).

The two most frequently used anticholinergic drugs are oxybutynin and tolterodine (13). This study assessed the efficacy of oxybutynin and tolterodine in children with resistant enuresis.

## Material and Methods

In this randomized clinical trial, patients aged between 6 and 16 years suffering from primary enuresis, referred to the pediatric nephrology clinic at Bu Ali Hospital, were studied. All patients received counseling, which included recommendations such as reducing fluid intake, ensuring adequate urination before bedtime, promoting positive incentives, and avoiding verbal, physical, or emotional punishment.

**Inclusion criteria**: were as follows: age between 6 and 16 years, primary enuresis, absence of clinical, urine test, or ultrasound evidence of secondary enuresis, and no response to nasal desmopressin treatment.

**Exclusion criteria**: includes secondary enuresis, underlying genitourinary system anomalies, history of antidepressant or anticholinergic use within the past three weeks, failure to continue follow-up, drug allergies to antidepressants, and congenital heart disease.

A re-evaluation was performed after two weeks. Patients who responded to these supportive measures were advised to continue. For those who did not respond adequately, desmopressin treatment was initiated (with a daily dose of one puff per dose, titrated up to three puffs per day, depending on the clinical response). After six weeks, either oxybutynin or tolterodine was added for patients who did not respond to desmopressin.

Patients were randomly assigned to two groups for a three-month treatment period, either receiving tolterodine + desmopressin or oxybutynin + desmopressin. The dose for oxybutynin was set at 0.2 mg per kg body weight per night, while tolterodine was given at 0.5-4 mg per night. Initially, the minimum starting dose was administered, and the patients were monitored for responses. Patients who showed remission at any dose were considered responsive. For those who did not respond to the starting dose, drug doses were gradually titrated to higher doses up to the maximum dose. Patients who showed no response to the maximum dose were considered non-responsive or resistant. The goal was not to assess differences in patient doses. A cut-off of six weeks was set for improvement or resistance based on our references (14-17).

The outcome was determined based on the patient's response to treatment after six weeks, ascertained via telephone or in-person follow-up. The primary outcome was the number of wetting incidents per night, and the secondary outcome was the number of wetting incidents per week. These were compared to pre-treatment rates. The response rate was categorized into four groups: 1-unchanged, 2- less than 50% improvement, 3-more than 50% improvement, and 4- complete recovery.

Although the medications used did not introduce new side effects, common side effects of oxybutynin and tolterodine, such as constipation, blurred vision, hot flashes, and skin allergies, were observed. The sample size was determined based on Lundmark's work (18), with an α= 0.05 and ß= 0.2, resulting in a total population size of 55.5. Data analysis was performed using SPSS software version 24, and results were presented as percentages, averages, quarters, and minimum and maximum values. Chi-square or Fisher's exact tests were used for comparing variables that required matching. A significance level of less than 0.05 was used as the criterion for statistical significance.

Ethical considerations included maintaining the confidentiality of participants' information and utilizing only known and routine enuresis treatment processes. The type of medication, possible side effects, and benefits were explained to patients or their parents, who then provided signed consent after reading through the information. Adherence to the Helsinki Legal Treaty's relevant clauses was ensured. The clinical trial was registered in the Iranian clinical trial registration system (IRCT20141224020419N3).

## Results

In this study, 68 children and adolescents suffering from desmopressin-resistant enuresis who met the inclusion criteria were randomly divided into two groups. The first group, consisting of 30 patients, received desmopressin + oxybutynin (oxybutynin group), and the second group, consisting of 38 patients, received desmopressin + tolterodine (tolterodine group). The mean age of all patients was 89 ± 27 months, with 56% (38 patients) being male. The demographic characteristics of the participating patients are listed in Table 1.

**Table 1:** Some basic and demographic data for 68 children with enuressis in two groups

	Group 1 (Desmopressin+Oxybutynin)		Group 2 (Desmopressin+Tolterodin)		Total		P-value

	Mean±SD	Median (25^th^-75^th^)	Mean±SD	Median (25^th^-75^th^)	Mean±SD	Median (25^th^-75^th^)	
Age (months)	86±27	79(64-111)	91±28	88(68-115)	89±27	83(65-112)	0.367
Male sex	17 (57)		21 (55)		38 (56)		0.909
N (%)							
Positive family	5 (17)		11 (29)		16 (24)		0.239
history, No (%)							
BUN (mg/dl)	19±4	20(15-21)	17±5	19(15-20)	19±5	19(15-21)	0.386
Cr (mg/dl)	0.63±0.14	0.57	0.65±0.14	0.67	0.64±0.14	0.63(0.50-	0.509
		(0.50-0.73)		(0.54-0.78)		0.76)	
Na (meq/l)	135 ±4	135	138 ±7	136	137 ±6	136	0.362
		(132-138)		(135-139)		(134-139)	
K (meq/l)	3.9±0.5	3.8(3.5-4.5)	3.9±0.4	4.0(3.5-4.3)	3.9±0.5	3.8(3.5-4.4)	0.719
Urinary	1.018±0.009	1.018	1.017±0.008	1.016	1.018±0.008	1.016	0.972
specific gravity		(1.010-1.028)		(1.010-1.023)		(1.010-1.023)	

No significant differences were observed between the two groups regarding sex, age, and family history of enuresis.

After six weeks, the clinical response to treatment was obtained via telephone or in-person follow-ups. Of the patients, 20 (29%) showed no response to the newer medications, while 11 (16%) experienced complete recovery. Another 11 (16%) reported less than 50% improvement, and 26 (38%) demonstrated more than 50% improvement. The primary therapeutic response was assessed based on the weekly wetting incidents compared to pre-treatment rates.

Prior to treatment, the average number of wet nights per week was seven for both groups. After one month of treatment, this average decreased to 3.3 ± 2.5 nights for the oxybutynin group and 4.2 ± 2.4 nights for the tolterodine group. However, the difference between the two groups was not statistically significant (P = 0.872).

Table 2 displays the four levels of response in both groups, revealing no significant difference between the two groups.

**Table 2:** Response to therapy in two treatment groups of children with enuresis

Response	Group 1 (Desmopressin+Oxybutynin) No (%)	Group 2 (Desmopressin+Tolterodin) No (%)	Total No (%)
No response	7(23)	13(34)	20(29)
Less than 50% improvement	3(10)	8(21)	11(16)
Equal or > 50% improvement	14 (47)	12 (32)	26 (38)
Complete cure	6 (20)	5 (13)	11 (16)

In addition to the primary response criterion, other criteria were also evaluated. In one criterion, any level of improvement was considered a response, while a lack of improvement was deemed a treatment failure. Using this criterion, 23% of the oxybutynin group and 34% of the tolterodine group did not respond to treatment, with the difference between the two groups being statistically insignificant (Table 3).

**Table 3:** Different levels of responsiveness in two treatment groups of children with enuresis

Response		Group 1 (Desmopressin+Oxybutynin) No (%)	Group 2 (Desmopressin+Tolterodin) No (%)	P-value
Incomplete	No response	10(33)	21(55)	0.089
responsiveness	Any response	20(67)	17(45)	
Partial	<50% improvement	7 (23)	13 (34)	0.424
responsiveness	≥50% improvement	23 (77)	25 (66)	
Complete	No complete cure	24 (80)	33 (87)	0.518
responsiveness	Complete cure	6 (20)	5 (13)	

In another criterion, a treatment response of less than 50% was considered inadequate, while a response rate of 50% or more was considered an appropriate treatment response. As shown in Table 3, 37% of the oxybutynin group and over half of the tolterodine group did not respond well to treatment, although the difference was not statistically significant.

Finally, in the last criterion, the complete elimination of enuresis was expected. The continuation of enuresis, regardless of the number of nights per week, was considered an unsuccessful treatment response. According to this criterion, the rate of 100% recovery was higher in patients treated with oxybutynin (20%) than in those treated with tolterodine (13.2%), but this difference was not statistically significant.

**Figure 1:**
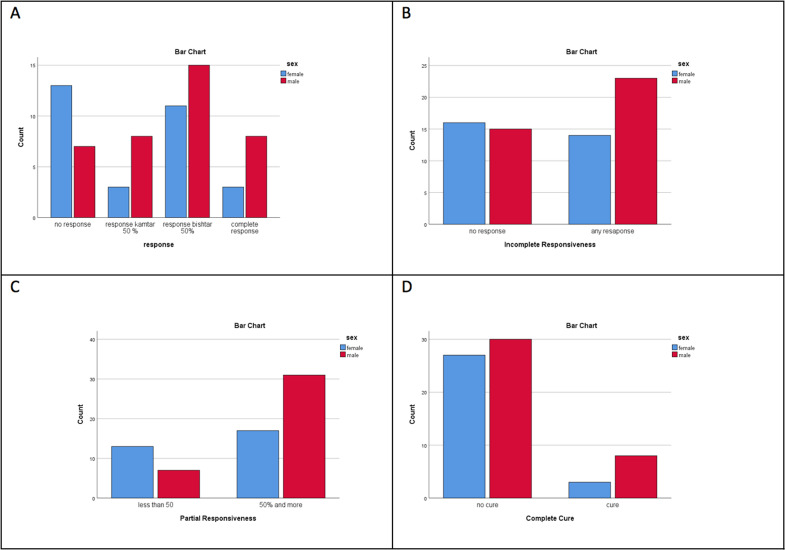
Different levels of responsiveness based on sex in children with enuresis. A; four level of responses (P=0.059), B; incomplete responsiveness (no response vs any response, P=0.258 ), C; partial responsiveness (<50% response vs >50% response, P=0.026 ), D; complete cure (complete cure vs any level of non-response, P=0.323)

The treatment responses of patients were also analyzed by gender. The only significant difference observed was in partial responses, with 13 out of 30 (43%) girls showing less than 50% improvement, compared to 7 out of 38 (18%) boys who demonstrated such improvement (P = 0.026 and Odds Ratio = 3.39). No other significant differences were found between the two sexes in terms of other response parameters (Figure 1).

The therapeutic response of patients to drugs was further evaluated based on family history. No significant differences were noted between groups with and without a family history in terms of treatment response across all studied parameters (Table 4).

**Table 4:** Response to therapy based on family history (FH) of enuresis

Response	Positive FH No (%)	Negative FH No (%)
No response	6(38)	14(27)
Less than 50% improvement	1(6)	10(19)
Equal or > 50% improvement	4 (25)	22 (42)
Complete cure	5 (31)	6 (12)
Total	16 (100)	52 (100)

None of the patients reported symptoms such as drowsiness, blurred vision, dizziness, headache, nausea, seizures, vomiting, diarrhea, weight gain, palpitations, dry mouth, fever, chills, or cough as treatment complications. A single instance of constipation was reported in each group, which was not statistically significant (P = 0.559). One urinary retention and two allergic reactions were reported in the tolterodine group, with no such reports in the oxybutynin group. However, these differences were not statistically significant (P = 0.309, P = 0.559, respectively).

## Discussion

Nocturnal enuresis is a common clinical problem in children (19). The problem could easily diagnosed in screening and assessment of UTI and other clinical problems (20,21). In this study, the effects of two anticholinergic drugs, oxybutynin, and tolterodine, combined with desmopressin, were investigated in desmopressin-resistant enuretic patients. Over 70% of patients in our study demonstrated some degree of response to treatment, and if the benchmark for treatment response is at least 50%, this percentage reaches around 50%. Since non-treatment of this condition can still result in a 15% annual spontaneous recovery, adding one of these drugs can significantly improve.

In a study by Seyfhashemi, patients with primary enuresis were divided into three treatment groups, each receiving one of three drugs: desmopressin, oxybutynin, or imipramine. Their criterion for a successful drug treatment response was complete remission for two weeks. Overall, 65% of patients in this study fully responded to treatment, with the highest response rate of 71% observed among oxybutynin recipients. However, no statistically significant differences were found between the three groups (22). If we were to adopt this criterion, a complete response would have been observed in only 20% of our patients. It is important to note that all of our patients were resistant to desmopressin, while in the aforementioned study, patients were at the first line of treatment.

In a study by Ghasemi, 66 patients with primary enuresis were divided into two groups, one receiving desmopressin and the other oxybutynin. The group receiving oxybutynin had significantly lower success rates in enuresis treatment. Nocturia persisted in 92.6%, 63%, and 48.1% of patients in the first, third, and sixth months post-treatment. Ghasemi concluded that oxybutynin was less effective in treating enuresis (23).

In a parallel study, Lee examined 158 children and adolescents aged 5-15 years with enuresis. These subjects were divided into three groups, with each group receiving either desmopressin, imipramine, or a combination of desmopressin and imipramine. The best response was observed in the combined treatment group, with the number of wet nights during a two-week treatment period decreasing from 13.3 nights to 3.7. In comparison, the desmopressin group went from 12 to 4 nights, and the imipramine group saw a slight increase from 9.2 to 9.3 nights (24).

In our study, the focus was on patients resistant to desmopressin. When oxybutynin was added to their treatment, a complete recovery was observed in 20% of patients, while a significant improvement (more than 50%) was noted in another 43% of patients' wet nights. Whether a 20% improvement is significant compared to a 15% annual spontaneous improvement depends entirely on the individual patient's circumstances and family situation.

The second group in our study received a combination of desmopressin and tolterodine. In a related study, Kazemi compared a combined treatment regimen of desmopressin and tolterodine with a therapy combining desmopressin and a placebo. Response rates were categorized into four groups: complete response, greater than 90% response, the relative response of 50-90%, and unsuccessful treatment (less than 50% reduction of wet nights). In the combined treatment group, a complete recovery rate of 54% and a partial recovery rate of 42% were observed, with only one patient (2%) failing to respond to treatment. In comparison, the desmopressin and placebo groups reported rates of 32%, 64%, and 4%, respectively. This difference was somewhat significant (P = 0.041). The average number of wet nights per week before treatment in the combined treatment group was 3.89 ± 1.19, which decreased to 1.05 ± 0.75 after treatment, a statistically significant reduction (P <0.001) (25).

Neveus conducted a comparative study of tolterodine, imipramine, and placebo in desmopressin-resistant children aged 6-13 years suffering from enuresis. Over the final two weeks, the average number of dry nights during two weeks of treatment for imipramine, tolterodine, and placebo was 11.0 ± 3.9, 10.4 ± 3.9, and 7.8 ± 5.1, respectively, showing a significant difference (P <0.001). Among the groups, only the imipramine group demonstrated a statistically significant response compared to placebo (26). Even though our patients initially showed resistance to desmopressin, this drug did not provide any new advantage in terms of complete recovery.

Austin evaluated 34 patients with desmopressin-resistant enuresis, dividing them into two groups. Eighteen patients received tolterodine in addition to desmopressin, while 16 patients were treated with desmopressin and a placebo. In the placebo group, only one patient (6%) exhibited a complete response, four patients (25%) showed a partial response, and 11 (69%) patients did not experience successful treatment. In contrast, the complete and relative responses in patients treated with tolterodine were 3 (17%) and 5 (28%), respectively, indicating a significant difference (27).

Ultimately, our study compared two treatment methods, desmopressin + tolterodine and desmopressin + oxybutynin, but no significant difference was found in treatment response. The study represents a novel approach. A literature search was conducted to find research papers on the combination of desmopressin and oxybutynin/tolterodine, but only one article was found.

Azarfar conducted a study involving 59 patients with enuresis who were treated under two regimens: desmopressin + oxybutynin and desmopressin + tolterodine. One month after treatment, 72% of the oxybutynin group and 83% of the tolterodine group reported complete recovery, with no significant difference between the two groups. These percentages were 45% and 87% three months after treatment. A significantly higher treatment response was observed in the tolterodine group during this period (P <0.001) (28).

The key distinction between the aforementioned study and ours is that the study participants received initial treatment, while our study focused on patients with refractory enuresis. Numerous studies have been conducted on patients with refractory enuresis, but the effects of anticholinergic therapy have received relatively little attention. Berkenwald integrated low-dose oxybutynin therapy with desmopressin in 61 patients with desmopressin-resistant enuresis and increased the dose of Oxybutynin to 7.5-10 mg if there was no response. In this study, the initial response rate over a two-week period of monotherapy with oral desmopressin was 59%, and 25 patients were unresponsive to monotherapy. Oxybutynin, at a dose of 5 mg, was added to the treatment regimen of these 25 patients. As a result, 17 patients (68%) responded positively to the treatment. For the remaining eight patients, an additional 2.5 mg dose of oxybutynin was added every two weeks until a maximum of 10 mg or a complete response was achieved. Six out of these eight patients (75%) responded favorably to this protocol. Overall, they reported a 96% success rate with monotherapy and titration (29). In a crossover study, Lundmark examined 18 patients with desmopressin-resistant enuresis over three periods of four weeks. He prescribed a course of reboxetine, desmopressin, and finally, only a placebo in the third course. During the first period, one patient achieved a complete response, and three patients exhibited a moderate response (at least 50%). In the second period, no patient achieved a complete response, but a relative response was observed in five patients. Lastly, no response was observed in the placebo group. Despite reporting a significant difference between the three groups, the author could not conclusively determine the efficacy of this treatment response (30)

In our study, a complete response, marked by a total absence of wet nights, was observed in only 11 patients (16%). However, more than 70% of patients exhibited some degree of response to the treatment, and in over 70% of patients, this response was substantial (at least 50%). What practical significance does this finding hold? It is crucial to underscore that clinical intervention should not be overly rigorous for treatment, given that this condition is fundamentally akin to a developmental delay that resolves on its own in more than 90% of cases. Once we have diagnosed primary enuresis through our examinations, it is our responsibility to consult with the parents. If enuresis does not pose issues in the patient's daily life and if the parents express no desire for medication or other treatments, no undue pressure for treatment is applied. However, suppose the parent or patient finds the condition problematic and perceives the need for medical intervention. In that case, we, as counselors, must articulate the possibility of spontaneous disease recovery, the likelihood of recovery with various medications and treatments, and the potential side effects associated with each, and assist them in their decision-making process. The aforementioned data serve as an excellent guide for such decisions.

No documented information about a synergic effect was found in the literature. If any relation exists, it is an advantage of the drug, but the mechanism of response was not the objective of our study. The design of our study cannot answer this question. Our study had certain limitations. The small sample size was a relative constraint. Our patients were selected from children resistant to desmopressin, which may be atypical for some clinical settings. Finally, the ambiguous evidence regarding synergic drug therapy posed another limitation.

In conclusion, in desmopressin-resistant patients, the addition of anticholinergic drugs results in a complete treatment response of 16% and a significant response in over half of the patients. No difference was observed in the treatment response between Oxybutynin and Tolterodine.
